# Nanofibers with homogeneous heparin distribution and protracted release profile for vascular tissue engineering

**DOI:** 10.3389/fbioe.2023.1187914

**Published:** 2023-06-22

**Authors:** Hongmei Zhang, Qilu Zhang, Juan Du, Tonghe Zhu, Dian Chen, Feiying Liu, Yang Dong

**Affiliations:** ^1^ Department of Orthopedics Surgery, Shanghai Sixth People’s Hospital Afffliated to Shanghai Jiao Tong University School of Medicine, Shanghai, China; ^2^ School of Chemistry and Chemical Engineering, Shanghai University of Engineering Science, Shanghai, China; ^3^ School of Textiles and Fashion, Shanghai University of Engineering Science, Shanghai, China; ^4^ Department of Cardiothoracic Surgery, Shanghai Children’s Medical Center, Shanghai Jiao Tong University School of Medicine, Shanghai, China; ^5^ School of Biotechnology, East China University of Science and Technology, Shanghai, China

**Keywords:** heparin, electrospun vascular grafts, mechanical compliance, thrombosis, patency rate

## Abstract

In clinic, controlling acute coagulation after small-diameter vessel grafts transplantation is considered a primary problem. The combination of heparin with high anticoagulant efficiency and polyurethane fiber with good compliance is a good choice for vascular materials. However, blending water-soluble heparin with fat-soluble poly (ester-ether-urethane) urea elastomer (PEEUU) uniformly and preparing nanofibers tubular grafts with uniform morphology is a huge challenge. In this research, we have compounded PEEUU with optimized constant concentration of heparin by homogeneous emulsion blending, then spun into the hybrid PEEUU/heparin nanofibers tubular graft (H-PHNF) for replacing rats’ abdominal aorta *in situ* for comprehensive performance evaluation. The *in vitro* results demonstrated that H-PHNF was of uniform microstructure, moderate wettability, matched mechanical properties, reliable cytocompatibility, and strongest ability to promote endothelial growth. Replacement of resected abdominal artery with the H-PHNF in rat showed that the graft was capable of homogeneous hybrid heparin and significantly promoted the stabilization of vascular smooth muscle cells (VSMCs) as well as stabilizing the blood microenvironment. This research demonstrates the H-PHNF with substantial patency, indicating their potential for vascular tissue engineering.

## 1 Introduction

In recent years, the incidence of arteriovenous fistula and peripheral vascular diseases are increasing year by year, and vascular bypass transplantation is the most important treatment method (Martinez-Gonzalez et al., 2017; Caliskan et al., 2020). Therefore, there is an urgent need for tissue engineered vascular grafts, especially small-diameter vascular grafts. The main challenge to the limited application of small-diameter artificial blood vessels is that early thrombosis, lumen stenosis, intimal hyperplasia and low long-term patency rate (Tatterton et al., 2012; Seifu et al., 2013; Zhu et al., 2021a; Zhuang et al., 2021; Li et al., 2023). In addition, the mechanical compatibility of small-diameter vascular grafts should be the focus of attention (Zhu et al., 2020; Zhu et al., 2021a; Zhi et al., 2022). Therefore, to achieve a long-term patency rate, the ideal small-diameter vascular grafts should have proper mechanical properties, outstanding blood compatibility and biocompatibility (Stahl et al., 2023).

Biomedical polymeric nanofibers present unique advantages and wide applications in the field of vascular tissue engineering due to three-dimensional biomimetic extracellular matrix (ECM) structure, adjustable physical and mechanical properties, stable biocompatibility as well as easy processing (Fathi Karkan et al., 2019; Nemati et al., 2019; Purushothaman et al., 2020). Poly (ester-ether-urethane) urea (PEEUU) is a biocompatible polyurethane derivative, which present excellent physical and mechanical properties and special microphase separation structure (Asadpour et al., 2018; Zhu et al., 2022). The soft and hard segments form the key surface structure of hydrophilic and hydrophobic alternating blocks in PEEUU, which not only conforms to Okano’s hypothesis of the relationship between anticoagulant blood and material surface structure, but also has a similar structure as in human blood vessels (He et al., 2010). But pure PEEUU still cannot achieve the ideal blood compatibility and histocompatibility. Therefore, through continuous exploration, previous researchers have developed a series of methods or technologies to improve the anticoagulation of polyurethane, which included the improvement of the hydrophilicity of polyurethane, the constructing of ionic polyurethane surface, surface grafting or blend using bioactive macromolecules.

Among many anticoagulant units, poly (ethylene glycol) (PEG) is often selected as the coating component grafting on the surfaces of vascular lumen to reduce the adsorption rate of plasma protein and platelet adhesion (Tseng and Park, 1992; Altankov et al., 2000). Kim et al. reported a method for grafting end-sulfonated PEG on the surfaces of polyurethane membranes. The results of blood compatibility showed that the PEG-grafted membrane significantly reduced the adhesion of platelets and prolonged the APTT time (YoungKim et al., 2003). Zhu et al. reported a functionalized nanofibers vascular grafts with PEG and heparin via the sequential covalent grafting method. The grafted PEG and heparin synergistically decreased the water contact angle of nanofibers as well as increased the capability for preventing platelet deposition (Zhu et al., 2021b). Lee et al. reported a modification method of PEO-PPO-PEO triblock copolymer mixed with polyurethane and then formed a film by solvent volatilization. The results revealed that the antiplatelet adhesion of the modified polyurethane was improved and increased with the increase of the proportion of PEO chain segments in the block copolymer (Lee et al., 1998). The surface modification using zwitterion is also an alternative method to improve the anticoagulation of materials (Ukita et al., 2019; Wang et al., 2020; Zhu Tianyu et al., 2021). The modified surface can reduce the adhesion of platelets on the surface of the material through electrostatic repulsion between negative charges. Yuan et al. also reported a surface grafting technique based on plasma treatment. Firstly, polyurethane membrane was treated with O_3_ plasma. Then, acrylic acid was grafted on the membrane. Finally, 1, 3-propyl sulfonolactone was grafted on the membrane by two methods. The blood compatibility test showed that the anticoagulation of polyurethane grafted with sulfonic acid anion group was significantly improved (Mokhtari and Kharazi, 2021). Among many bioactive macromolecules, heparin has excellent anticoagulant properties and is the most commonly used anticoagulant. After more than 40 years of continuous development, the immobilization technology of heparin is showing more and more improvement in medical field. Although heparin presented obvious anti-coagulation and anti-hyperplasia effects, the existing immobilization methods, such as physical blending, ionic bonding, and covalent bonding by solution blending or solution grafting, cannot solve the problems of fast release, easy inactivation, and structural changes of heparin, which may be due to its very short half-life and easy dissolution in water rather than in organic solvents (Serrone et al., 2013; Kevin, 2015; Yang et al., 2022).

In this work, we propose a novel small diameter vascular graft to solve the above mentioned problems that the uneven dispersion of heparin in vascular material. Based on our previous research on nanofibers for sustained drug release and vascular tissue regeneration, the optimized concentration of heparin, was combined with synthetic PEEUU elastomer, following hybrid PEEUU/heparin nanofibers prepared by homogeneous emulsion blending and electrospinning for exploring blood vessel repair effect and mechanism. We evaluated the physicochemical properties, microscopic morphology, and cytocompatibility of hybrid PEEUU/heparin nanofibers, and then detected the activity of promoting tissue regeneration and inhibiting thrombosis and intimal hyperplasia. Finally, the abdominal aortic defect model of rats was used to evaluate the effect of hybrid PEEUU/heparin nanofibers tube.

## 2 Materials and methods

### 2.1 Materials

Poly (ester-ether-urethane)urea elastomer (PEEUU, Mw = 9.2 × 10^4^) were synthesized via a two-step solution polymerization in synthesis laboratory of functional polymer of Shanghai University of Engineering Science. Heparin sodium powder (150 U/mg) (Hep) were purchased from Sigma-Aldrich Trading Co., Ltd. (Shanghai, China). Human umbilical vein endothelial cells (HUVECs) for *in vitro* experiments were obtained from Shanghai Cell Bank of Chinese Academy of Sciences (Shanghai, China). Cell counting kit (CCK-8) was obtained from Sigma-Aldrich Trading Co., Ltd. (Shanghai, China). Dulbecco’s modified eagle medium (DMEM), Fetal bovine serum (FBS), and antibiotic-antibacterial medicine (penicillin/streptomycin) were purchased from Hyclone Trading Co., Ltd. (Shanghai, China). Hexafluoroisopropanol (HFIP, purity ≥99.2%) was obtained from Shanghai Darui Fine Chemicals Co., Ltd. (Shanghai, China). Unless otherwise specified, all the above reagents were used directly. All the materials were used as received, except where mentioned otherwise.

### 2.2 Preparation of nanofibers tubes

To prepare hybrid PEEUU/heparin nanofibers tubular graft (H-PHNF), PEEUU was dissolved in 10 mL of HFIP to form a mixture with 8.0 w/v% concentration with stirring until clarified. 0.01 g of heparin sodium (150 U/mg) was dissolved in three drops (about 0.2 mL) of distilled water until clarified, following addition of above-clarified PEEUU/HFIP mixture forming electrospinning solution with a relative mass fraction W_Hep_/W_PEEUU_ of 1.25% under vigorous stirring at room temperature for 72 h, respectively. Then, the 10 mL of the above prepared mixture solution were electrospun to generate nanofibers with a constant speed of 1.0 mL/h and a voltage of 12 kV.

Electrospinning solution for preparing PEEUU nanofibers (PNF) was prepared according to the following protocol: Synthetic 0.8 g PEEUU was dissolved in 10 mL HFIP formed a mixture with 8% concentration with stirring until clarified. To preparing the electrospinning solution for fabricating the blend PEEUU/heparin nanofibers (B-PHNF): Synthetic 0.8 g PEEUU was dissolved in 10 mL HFIP formed a mixture with 8% concentration. After stirring until clarified, quantitive heparin sodium powder (Hep) was added in the above-clarified mixture forming electrospinning solution with a relative mass fraction (W_Hep_/W_PEEUU_) of 1.25%.

The parameters to generate nanofbers showed in Table 1, respectively. The as-electrospun nanofibers were collected onto a stainless steel bar (2.0 mm diameter, 100.0 mm length, rorated at 200 rpm) or a flat aluminum foil board located 14 cm from the capillary to form nanofibers tubes or nanofibers mats, namely, PNF, B-PHNF, and H-PHNF, respectively. Then nanofibers tubes and nanofibers mats were vacuumed in a desiccator for 48 h to remove redisual HFIP.

**TABLE 1:** Settings of PEEUU, heparin sodium dosage, and HFIP bulk in control and experimental groups, respectively.

Samples	PEEUU (g)	Heparin sodium (mg)	HFIP (mL)
PNF	0.8	0	10
B-PHNF	0.8	10	10
H-PHNF	0.8	10	10

### 2.3 Characterization and testing

The morphology and surface structure of nanofibers and nanofibers tubes were carried out using a scanning electron microscope (SEM, Phenom XL, Netherlands) operating with sputter gold plating for 35 s at 5 mA at an accelerating voltage of 10 kV. A contact angle measuring device (JC 2000D 2A, Shanghai Zhongchen Digital Technology Equipment Co., Ltd., China) was used to test the wettability of nanofibers tubes. To test this, 0.02 mL deionized water was added to the sample and three different positions of the sample were taken to measure the water contact angle and calculate the average value. Image-J (United States) was used to determine the diameter of nanofibers, inner diameter and wall thickness, and the pore diameter of nanofibers tubes.

The ethanol displacement method was applied to confirm the porosity of nanofibers tubes. The dried tubes were dipped in absolute ethanol, and the bubbles were removed. The porosity was determined by Eq. 1:

$$Porosity\;\left(\%\right)=\frac{V1-V3}{V2-V3}\times 100\%$$ (1)

where *V*_*1*_ expresses the volume of known ethanol; *V*_*2*_ stands for the bulk volume of the ethanol impregnated nanofibers tubes and ethanol; *V*_*3*_ is the volume after removed the nanofibers tubes.

High-precision tensile testing machine (HY-025CS, Shanghai Hengyu Instrument Co., Ltd., China) with a transducer with a load range of 0–200 N was employed to testing the mechanical properties of nanofibers tubes in wet conditions at room temperature strictly according to ISO 7198: 1998. Each sample was cut into a cylindrical ring with length × inner diameter × wall thickness = 10.0 mm × 2.0 mm × 0.4 mm, then soaked in 0.01 M phosphate buffer (PBS, pH = 6.8) for 24 h. Finally, tensile tests were investigated at room temperature with a stretching speed of 1.0 mm/min. The specimens were extended until breaking under tensile force and the tensile stress-strain curves were recorded. Each test was repeated five times during mechanical analysis. The tensile strength, elongation at tensile strength, the representative modulus, and first order equation fitting of stress and strain within the magnification of calculating range were calculated according to the results of stress-strain.

### 2.4 Heparin density and sustained release tests *in vitro*

The presence of heparin in fibers were verified via toluidine blue staining. Briefly, a 0.005% toluidine blue solution was prepared in hydrochloric acid (0.01 M) containing 0.2% NaCl. The samples were incubated in the prepared toluidine blue solution for 12 h and dried at room temperature for further observation. The heparin density in fibers were verified via prepared toluidine blue staining assay. Electrospun tubes (2.0 mm inner diameter, 15.0 mm length) were immersed in 50 mL aqueous solution of toluidine blue [500 μmol/L toluidine blue solution dissolved in Milli Q water with a pH value of 10 (adjusted with NaOH)] and incubated in a vapor-bathing constant temperature vibrator at 37°C with a vibrating speed of 100 rpm for 12 h. Samples were then washed with dilute NaOH (pH = 10) five times followed by immersing in 50 mL aqueous solution of acetic acid with a concentration of 50% at 37°C with a constant vibrating speed of 100 rpm for 30 min. The OD value of toluidine blue released from fibers was read at 633 nm using a microplate reader (MK3, Thermo, United States).

The nanofiber samples for sustained release were placed in a constant temperature vibrator at 37°C (100 rpm). At the set time points, 3 mL of release solution was removed, while 3 mL of fresh PBS medium was added. Three test samples were set for each group. The cumulative release amount of heparin was calculated according to the standard heparin absorbance concentration at 260 nm. The percentage of accumulated release can be calculated by Eq. 2:

$$ARP\;\left(\%\right)=\frac{C\times 30+\sum W}{m\times R\times 1000}\times 100\%$$ (2)

C-the concentration of heparin, μg/mL; ∑W-the mass of heparin accumulated release, μg; m-the mass of nanofibers tubes, mg; R-the percentage of drug within nanofiber.

### 2.5 Cell culture and blood compatibility tests *in vitro*

Human umbilical vein endothelial cells (HUVECs) were used to evaluate the activity of host cells, which were co-culture with PNF, B-PHNF, and H-PHNF nanofibers mats, respectively. HUVECs were obtained from Shanghai Cell Bank of Chinese Academy of Sciences (Shanghai, China) and cultured with growth medium consists of dulbecco’s modified eagle medium (DMEM), 10% fetal bovine serum and 1% penicillin/streptomycin. The cell viability of HUVECs were tested by using the Cell Counting Kit-8 (CCK-8). The cells were cultured in PNF, B-PHNF, and H-PHNF nanofibers mats for 1 day, 3 days, and 5 days, respectively. Detailed procedures are available in the Supplementary Material.

Fresh blood, which was drawn from the marginal vein, and 3.2% sodium citrate solution in a volume ratio of 9:1 (v/v) were collected using a plastic vacuum blood collection tube (2.7 mL, Becton Dickinson, United States), containing 3.2% sodium citrate solution. All animal experimental protocols are in accordance with the policy of the Institutional Review Board for Human Investigations at Shanghai Sixth People’s Hospital Afffliated to Shanghai Jiao Tong University School of Medicine. Detailed procedures are also available in the Supplementary Material (Mokhtari and Kharazi, 2021).

### 2.6 *In vivo* transplants to replace the abdominal aorta of rats

All rats were obtained from Shanghai Slaccas Experimental Animal Co., Ltd. (Shanghai, China) and all experimental schemes are in agreement with the requirements of the Institutional Animal Care and Use Committee (IACUC) of Shanghai Sixth People’s Hospital Afffliated to Shanghai Jiao Tong University School of Medicine and accepted by IACUC. Ethical principles were followed throughout the experiment. All experimental plans were proceeded in conformity of the Animal Management Regulations of China (1988 and revised in 2001, Ministry of Science and Technology).

Sixty-three male Sprague-Dawley rats (age about 10 weeks; weighing approximately 300 g; 18 rats for standby application) were used as the abdominal aortic replacement models in the research. A 7.0 mm long defect of the abdominal aorta was created and replaced by nanofibers tubes (2.0 mm inner diameter, 7.0 mm length), and then the nanofibers tubes was end-to-end sutured to the abdominal aorta. Performance of the nanofibers tubes graft was evaluated on first, second, and fourth week after surgery in total of five animals per group for each time point.

At predetermined time points, rats were anesthetized by intraperitoneal injection of pentobarbital (3.5 mg/100 g; Nembutal). A color Doppler ultrasonography platform (GE LOGIQ 9, GE Medical Systems, United States) was used to acquire images to evaluate the patency rate of vessels. Then rats were sacrificed after injecting an overdose of isoprene barbiturate from the ear margin vein. Implanted nanofibers tubes were retrieved and analyzed by H&E staining, Masson’s trichrome staining, Van Gieson, Saffron O, Von Kossa, and immunofluorescent staining. Detailed procedures are available in the Supplementary Material.

### 2.7 Statistical analysis

Data were presented as mean ± standard deviation. All data were analyzed by one-way ANOVA with Tukey’s *post hoc* tests. *p*-values <0.05 (∗) were deemed to be statistically significant. ∗ indicates *p* < 0.05, ∗∗ indicates *p* < 0.01, ∗∗∗ indicates *p* < 0.001.

## 3 Results and discussion

Synthetic vascular grafts perform satisfactorily as large-diameter (e.g., aortoiliac) arterial substitutes but commonly fail when employed in small-diameter applications (Wang et al., 2019). To date, clinically available expanded poly (tetrafluoro-ethylene) (ePTFE) or poly (ethylene terephthalate) (PET) vascular grafts are suboptimal for reconstructing small-diameter (inner diameter ≤6 mm) arteries, owing to thrombosis in early and restenosis in late stage (Lim et al., 2021; Roina et al., 2022). Moreover, ePTFE or PET vascular prostheses connected to human artery cannot swell when subject to the blood pressure owing to their poor elasticity, and thus also result in the decline of blood flow rate and the formation of thrombosis. Some previous research reported that polyurethanes were being applied in medical devices because they have the capability to tolerate contractile forces that originate during the cardiac cycle without undergoing plastic deformation or failure, and the capability to imitate the behaviors of different tissues (Rahimi and Mashak, 2013; Uscategui et al., 2018; Kelly Navas-GomezValero and Valero, 2020). More importantly, the mechanical compliance and other properties of polyurethane can be further improved by structural design and functional modification (Zhang Zhenyan et al., 2022). Therefore, polyurethane may be an optimal candidate.

Heparin is the most widely used and reliable anticoagulant that can enhance the patency and inhibit the thrombus. A lack of heparin leads to blood clotting, while an excess of heparin causes bleeding. (Kevin, 2015). Furthermore, the half-life of heparin is less than 1 h in circulation (Yang et al., 2022). Thus, maintaining localized concentration and homodisperse of heparin at the target site for long time is essential for implant revascularization. In this study, a hybrid electrospun BEPU/heparin nanofibers tubular graft compound with optimized constant concentration of heparin by homogeneous emulsion blending, were prepared for replacing rats’ abdominal aorta *in situ* for comparing with nanofibers tubular graft, which were prepared via resolution after blending of solute.

### 3.1 Microstructure and mechanical properties

**FIGURE 1:**
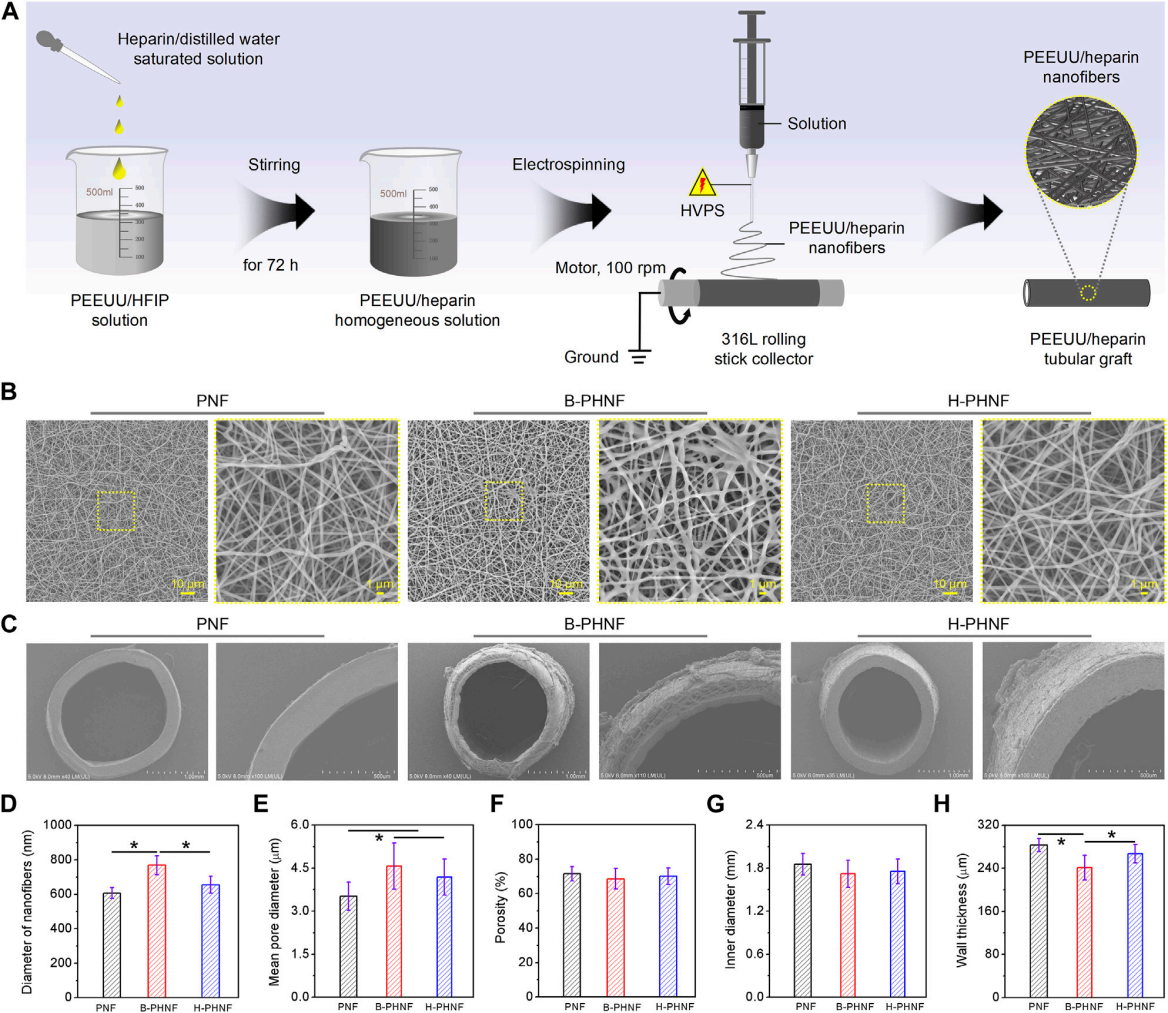
**(A)** The preparation diagram of PEEUU nanofibers tubular graft with heparin homogeneous distributions (H-PHNF); **(B, C)** SEM images of the lumen surface and the cross sections of PNF, B-PHNF, and H-PHNF, respectively; **(E–G)** Fibers diameters of the lumen surface, mean pore diameters, porosity, inner diameters, and wall thickness of PNF, B-PHNF, and H-PHNF, respectively. (Data are representatives of independent experiments and all data are given as mean ± SD, *n* = 5; ∗*p* < 0.05).

We have compounded PEEUU/HFIP solution with heparin/distilled water solution, and then processed it into clarified PEEUU/HFIP/heparin/distilled water mixture according to the solvent compatibility of HFIP and water. PEEUU/heparin could be feasibly dissolved in HFIP/distilled water mixture to form a homogeneous hybrid solution for random electrospinning and collected by a 316 L rolling stick collector to obtain tubular grafts (Figure 1A). Similarly, control samples were also prepared in this way, except for the different electrospinning solution.

The morphology of prepared tubular grafts are shown in Figures 1B,C. The SEM images of the lumen surface of PNF, B-PHNF, and H-PHNF, in Figure 1B, showing its nonwoven structure formed by random stacking of nanofibers with the average diameter of 607 ± 31 nm, 769 ± 55 nm, 655 ± 49 nm, respectively, (Figure 1D). The diameter of both PNF and H-PHNF group were finespun but evenly distributed, while agglomeration appeared in B-PHNF group. Correspondingly, the pore sizes of both PNF and H-PHNF group are smaller than those of B-PHNF groups, and the porosity is larger than that of B-PHNF group (Figures 1D–F).

Moreover, as shown in Figure 1C, the electrospun grafts could maintain its tubular shape after being peeled from the 316 L rolling stick collector. The statistical data of inner diameter showed that the average diameter of PNF, B-PHNF, and H-PHNF groups were 1.854 ± 0.152, 1.719 ± 0.189, and 1.756 ± 0.172 mm as well as the average wall thickness of PNF, B-PHNF, and H-PHNF were 283 ± 12, 241 ± 23, and 267 ± 17 μm, respectively (Figures 1G,H). Compared with B-PHNF group, the inner diameter sizes and wall thickness of both PNF and H-PHNF groups are larger than those of B-PHNF groups. We attributed these appearance to the fibers size and structure of grafts, and the distribution of heparin in fibers.

In the design of small-diameter artificial blood vessel, the first consideration should be the mechanical properties (Zhu et al., 2020). Due to its small diameter and thin wall, small-diameter artificial blood vessels are not strong enough to resist external forces. Therefore, the designed small-diameter artificial blood vessels should be able to resist certain pressure in the body without deformation. Moreover, the artificial blood vessels must have a burst pressure similar to or higher than that of human blood vessels, which have a burst pressure of more than 1700 mmHg.

**FIGURE 2:**
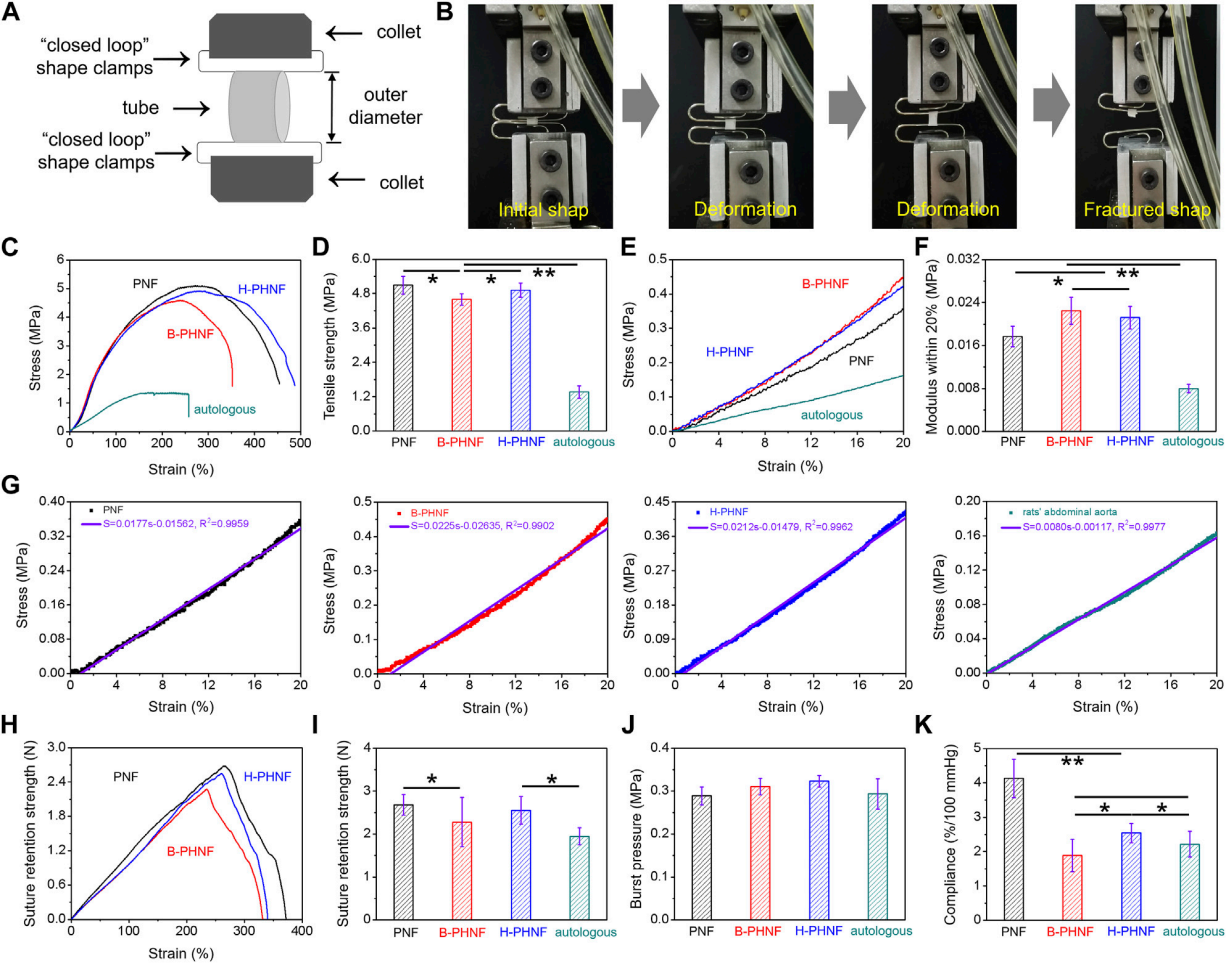
Mechanical properties of prepared nanofibers tubular grafts: presentative radial tensile results of PNF, B-PHNF, and H-PHNF under wet conditions. **(A)** Illustration of the measurement method for radial tensile properties of tubular grafts; **(B)** Digital photographs of initial shape and fractured shape of tubular grafts; **(C)** representative radial stress-strain curves; **(D)** Tensile strength; **(E)** The magnification of calculating range of 0–20% modulus; **(F)** Representative 0–20% modulus; **(G)** First order equation fitting of stress and strain within the magnification of calculating range of 0–20% modulus; **(H)** Suture retention strength-strain curves; **(I)** Maximum suture retention strength; **(J)** Burst pressure; **(K)** Complicance. (Data are representatives of independent experiments and all data are given as mean ± SD, *n* = 5; ∗*p* < 0.05, ∗∗*p* < 0.01).

We have measured the mechanical properties of nanofibers tubular grafts in wet conditions at room temperature strictly according to ISO 7198: 1998 using the uniaxial radial high-precision tensile testing machine as the schematic illustration in Figures 2A,B. As shown in Figure 2B, the zone of breaks of tubular graft is located in the middle of the sample, which means this fracture test is effective. Representative radial stress-strain curves and maximum tensile strength of PNF, B-PHNF, and H-PHNF are presented in Figures 2C,D, respectively. The results showed that the maximum tensile strength of PNF, B-PHNF, and H-PHNF are 5.096 ± 0.310 MPa, 4.596 ± 0.201 MPa, and 4.916 ± 0.251 MPa, respectively, which are significantly larger than 1.365 ± 0.211 MPa of rats’ abdominal aorta.

**FIGURE 3:**
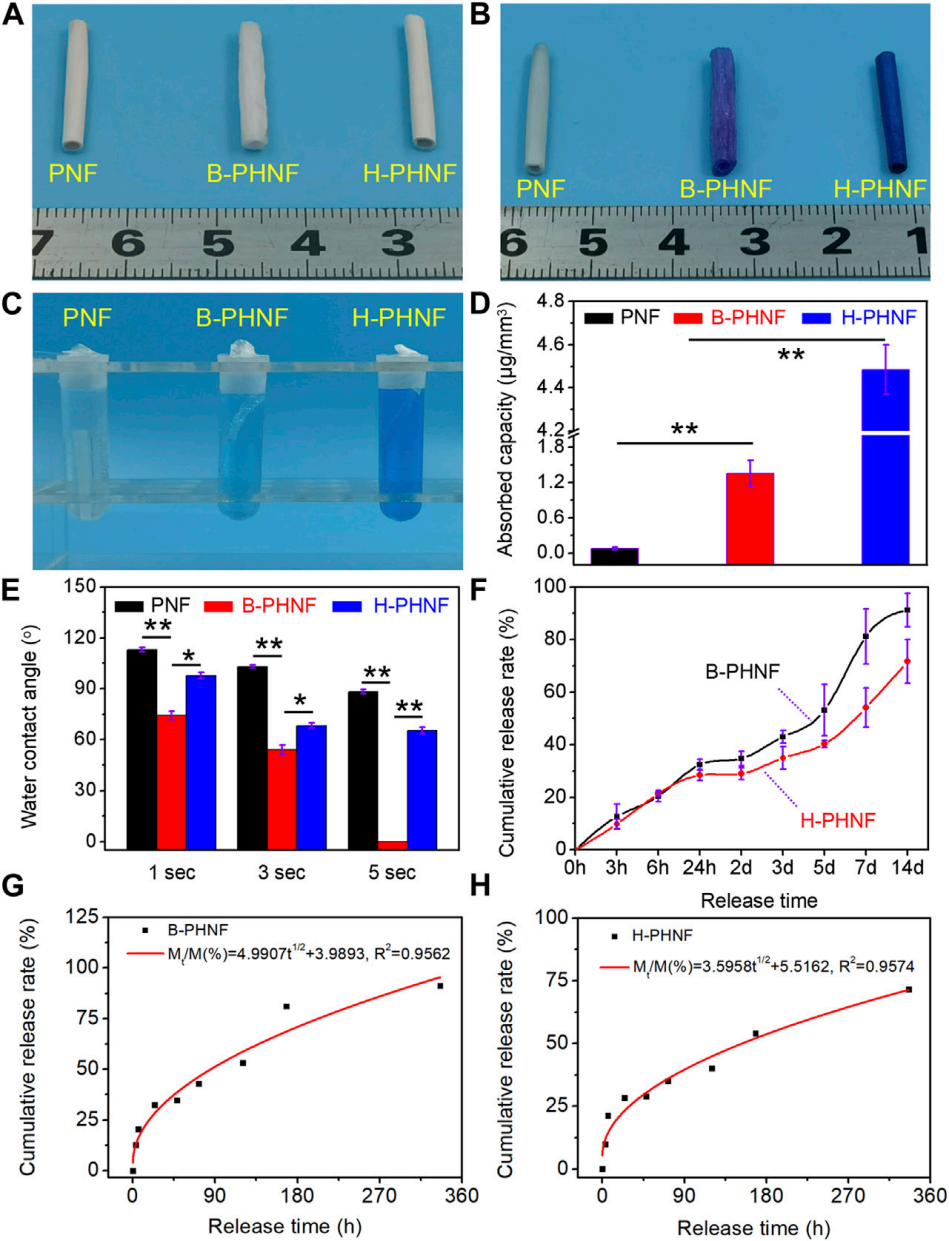
**(A)** The digital photos of PNF, B-PHNF, and H-PHNF, respectively; **(B)** Toluidine blue staining digital photos of PNF, B-PHNF, and H-PHNF in wet states, respectively; **(C)** Toluidine blue extract used acetic acid as treating agent; **(D)** Quantitative data of adsorption assays indicate in the amounts of toluidine blue in nanofibers tubular grafts; **(E)** Water contact angle at the 1, 3, and 5 s time point; **(F)** *In vitro* releases of heparin from B-PHNF and H-PHNF in pH = 6.8 PBS, respectively; **(G, H)** Higuchi equation fitting of cumulative release rate and release time of B-PHNF and H-PHNF, respectively. (Data are representatives of independent experiments and all data are given as mean ± SD, *n* = 5; ∗*p* < 0.05, ∗∗*p* < 0.01).

Natural blood vessels are viscoelastic bodies with viscoelastic characteristics such as creep and stress relaxation, which can be treated as elastic materials and have anisotropic nonlinear stress-strain relationship after sufficient preconditioning. As in Figures 2E–G, the representative radial stress-strain curves, representative 0–20% modulus, first order equation fitting of stress and strain within the magnification of calculating range of 0–20% modulus were calculated and fitted according to the dates in Figure 3E, respectively. It is clear that the modulus within the magnification of calculating range of 0–20% strain of H-PHNF significant increased compare to rats’ abdominal aorta (Figure 2F). The increase of modulus also proves that H-PHNF have good anti-deformation performance after implantation *in vivo*. In short, three electrospun tubular grafs showed reliable resiliency, while H-PHNF exhibited better initial elasticity, which was more suitable for application as a prosthetic blood vessel.

As shown in Figures 2H–J, the obtained burst pressure substantially reached those of both artery and saphenous vein which is the gold standard for bypass operation, whereas the suture retention strength was a little smaller than PNF but still quite larger than the rats’ abdominal aorta (around 0.293 MPa) for surgical operation.

Compliance is defined as the volume change in response to changes in blood pressure, and constitutes an important parameter that influences the short- and long-term patency of artificial blood vessels (Stahl et al., 2023). Especially in the early stage of transplantation, the mismatch of mechanical compliance between vascular grafts and autologous blood vessels is more likely to cause acute thrombosis at the anastomosis site. The compliance of PNF, B-PHNF, H-PHNF, and rats’ abdominal aorta were evaluated, respectively, as shown in Figure 2K. The compliance of both PNF and H-PHNF were larger than that of the other two groups. The compliance of H-PHNF is better than B-PHNF as well as rats’ abdominal aorta. PEEUU/heparin tubular graft through a combination of homogeneous emulsion blending technology and electrospinning technology exhibit prominent stable mechanical compliance due to the homogeneous dispersion of heparin in the electrospun fibers.

### 3.2 Surface wettability and heparin sustained release properties

We obtained tubular grafts with a length of 2.0 cm and an inner diameter of 2.0 mm (Figure 3A). The presence of as hybrid heparin was examined by toluidine blue staining and analyses exhibited color change from white to dark blue. Images of toluidine blue staining demonstrated that H-PHNF received more dye after staining (Figure 3B). This was in line with the quantitative study that the heparin-end groups had much higher absorbance than the other groups (Figures 3C,D). The results of water contact angle measurements are exhibited in Figure 3E. PNF showed its hydrophobic nature as the water drop well stood on its surface after it was dropped down and its contact angle was measured about 113° at the 1 s time point. When heparin molecule blended in the electrospun PEEUU fibers, the obtained B-PHNF exhibits higher hydrophilicity (the water contact angle is 74° at the 1 s time point), while the water contact angle of H-PHNF is 102° at the 1 s time point. We believe that the above phenomenon is not only closely related to the microstructure of the fiber, but also to the dispersion of the heparin molecules. Specifically, due to poor dispersion, more heparin molecules gathered on the surface of PEEUU fibers in B-PHNF, and these highly hydrophilic heparin molecules increased the roughness of the surface of PEEUU fibers. In general, the hydrophilicity of B-PHNF was stronger than that of H-PHNF.

In the process of drug release, the properties of drug release depend on complex physical and chemical phenomena (Zhu et al., 2021b). The factors controlling the drug release rate are not only the three-dimensional structure of the grafts, the interaction between molecules, the degradation rate of the material, the solubility of the drug, but also closely related to the dispersion of the drug in the polymer matrix. Figure 3F shows the cumulative drug release curve of heparin in B-PHNF and H-PHNF, where the *X*-axis is the release time, and the ordinate is the percentage of cumulative release in the total drug load. The release curves of heparin in B-PHNF and H-PHNF with similar drug concentration were compared. The release of heparin from both B-PHNF and H-PHNF mainly includes three stages: the initial drug release stage (0–24 h), controlled release stage (24 h–5 days), and late accelerated release stage (5 days∼). At the initial release phase, the released heparin mainly comes from the surface of the PEEUU fiber. Most of this heparin, which exposed to the surface of the fiber, is very vulnerable to the surrounding tissue fluid wash and rapid release. It is obvious that heparin in both B-PHNF and H-PHNF exhibited obvious initial release, and the cumulative release rate at 3 h reaches 12.69% and 9.84%, respectively. On the 14th day, the final cumulative release of heparin in both B-PHNF and H-PHNF reached 91.26% and 71.67%, respectively, which shows a clear difference. In short, heparin in H-PHNF was released at the slower rate than B-PHNF after 24 h, which presumably achieve long-term inhibiting thrombus and intimal hyperplasia effect. Moreover, according to the time square root equation proposed by Higuchi, the least square regression analysis was carried out for the release time t^1/2^ and the cumulative release amount M_t_/M (%). The fitting equations were M_t_/M(%) = 4.9907t^1/2^ + 3.9893 and M_t_/M(%) = 3.5958t^1/2^ + 5.5162, and the fitting correlation coefficients were 0.9562 and 0.9574, respectively. Figures 3G,H show that the release of heparin at the initial stage of B-PHNF and H-PHNF conforms to the Higuchi equation, and diffusion is the main mechanism of heparin release.

### 3.3 Hemocompatibility *in vitro*

**FIGURE 4:**
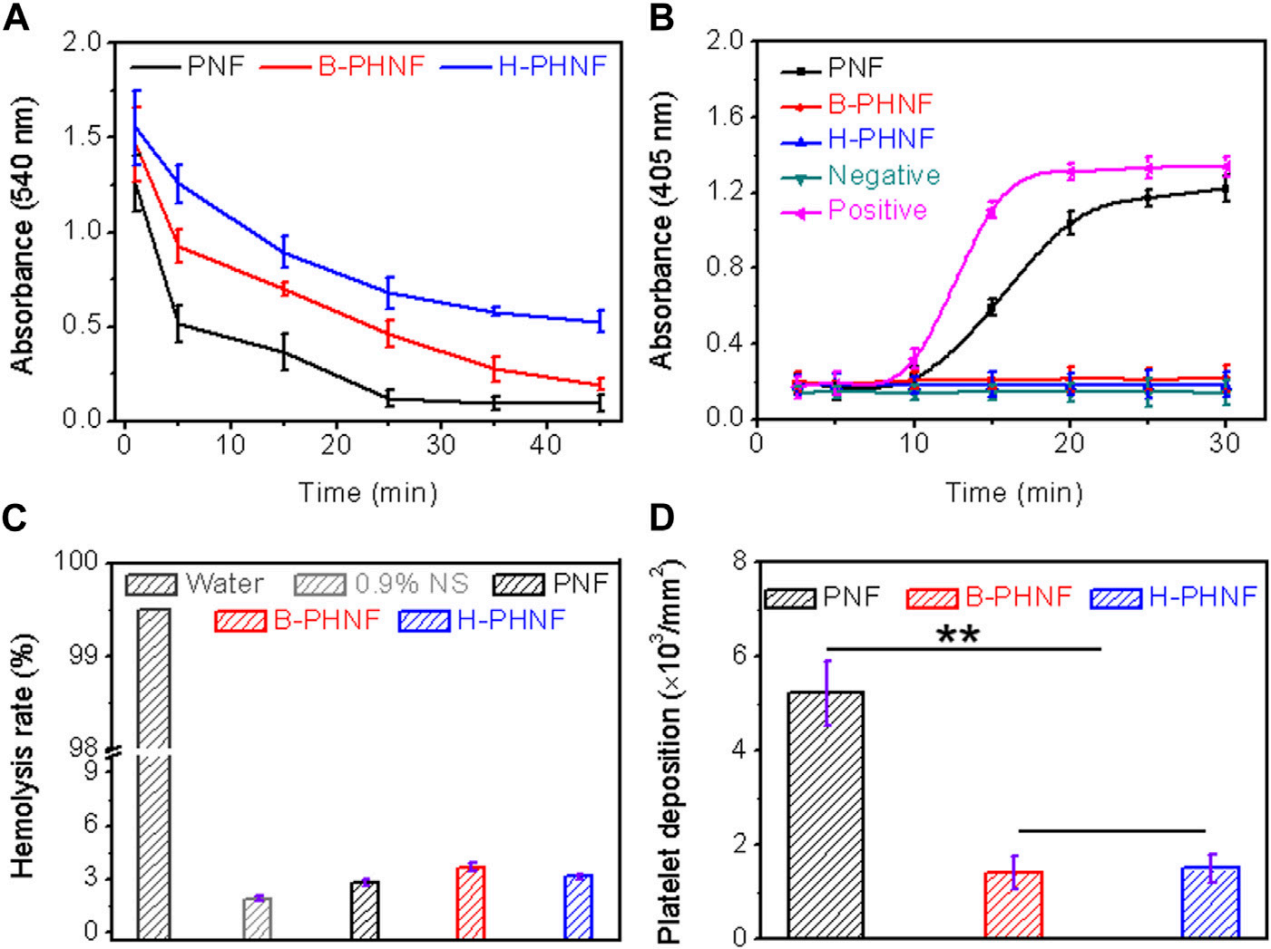
**(A)** Whole blood clotting time; **(B)** Plasma recalcification time; **(C)** Quantification of relative hemolysis rate; **(D)** Platelet deposition determined by lactate dehydrogenase assay. (For plasma recalcification time test, TCPs exposed to PPP with and without CaCl_2_ were used as positive control and negative control, respectively; For hemolysis test, water and 0.9% normal saline (NS) serve as positive and negative groups, respectively; Data are representatives of independent experiments and all data are given as mean ± SD, *n* = 5; ∗*p* < 0.05, ∗∗*p* < 0.01).

When the material comes into contact with blood, it causes platelet adhesion and activation of the clotting system. Since platelet and coagulation system work together to produce coagulation, the interaction between the two should be carefully considered in the design of blood compatible materials (Zhu et al., 2021b). After the contact between the isolated venous blood and the material, coagulation factors and the endogenous coagulation system are activated, and finally fibrin is generated and blood coagulation occurs. The time experienced in this stage is called coagulation time. Coagulation time can generally reflect the total coagulation ability of blood after contact with materials. After the whole blood incubation, the absorbance of the supernatant was measured at 540 nm, indicating the number of remaining erythrocytes, as shown in Figure 4A. Compared with the other two groups, the H-PHNF had the maximum absorbance at any time point. Under the same experimental conditions, the clotting time of both PNF and B-PHNF were significantly shorter, indicating that H-PHNF has anticoagulant properties, which may be due to its outstanding hydrophobic performance according to the contact angle results (Figure 3A).

All the clotting factors involved in endogenous clotting are supplied by plasma. When plasma comes into contact with the material, the surface charge activates the clotting factors in the plasma. In the presence of Ca^2+^, these clotting factors bind to Ca^2+^ to form prothrombin complexes, which activate and convert to active thrombin in the presence of thrombin and Ca^2+^. Subsequently, soluble fibrinogen in plasma is converted to insoluble fibrin by thrombin and Ca^2+^, which eventually leads to clotting. Plasma recalcium curve is a method used to characterize the endogenous coagulation system. Recalcium time refers to the time required for plasma coagulation after the removal of calcium source and the addition of Ca^2+^. The detailed anticoagulant mechanism is that Heparin forms a complex by binding with antithrombin Ⅲ, which accelerates the inactivation of coagulation factors and thus inhibits the formation of prothrombin kinase. In addition, heparin calcium also acts on already formed prothrombin kinases, thus providing a stronger anticoagulant effect. Figure 4B shows the plasma recalcium kinetic curves of PNF, B-PHNF, H-PHNF, positive control group, and negative control group. The positive control has the fastest clot formation time at 15 min, while the clot formation time of PNF was 20 min. Notably, B-PHNF, H-PHNF consistently kept a low absorbance and closed to the negative control without emerging the inflection point. These results indicate that the grafts complexed with heparin can significantly inhibit endogenous coagulation activation and improve the anticoagulation performance of the grafts.

When the material comes into contact with red blood cells, the degree of damage to red blood cells can be characterized by the hemolysis rate. The smaller the hemolysis rate of the material, the better the blood compatibility of materials. The hemolysis rate test results of PNF, B-PHNF, and H-PHNF samples are shown in Figure 4C. Figure 4C showed that the hemolysis rates of the three grafts were all less than 5% as specified in the ISO10993-4 standard, indicating that the three grafts were non-hemolysis materials with good blood compatibility, non-toxicity, and no obvious damage to erythrocytes. Moreover, both the unheparined and heparin-modified grafts had hemolysis rates of approximately 3%, indicating that heparinization did not affect the hemolytic properties of grafts.

The adhesion and activation of platelets can activate clotting factors and promote the formation of fibrin, which eventually leads to the formation of thrombus (Jordan et al., 2016). Therefore, the resistance against adhesion of platelets on the surface of materials is one of the important markers of the antithrombotic properties of materials. To compare the influence of compound heparin on the number of adherent platelets, the results of quantitative detection of the number of adherent platelets using the lactate dehydrogenase (LDH) kit are shown in Figure 4D. The number of platelet adhesion per unit area on the surface of the material is shown in Figure 4D. The platelets with obvious adhesion are on the surface of PNF, while the number of platelet adhesion is significantly reduced on both B-PHNF and H-PHNF surfaces due to the anticoagulation of heparin. In addition, there was no significant difference between the B-PHNF and H-PHNF groups, indicating that no matter which technique was used to combine heparin and PEEUU, the spatial conformation of heparin would not change significantly, let alone affect the anticoagulant effect of heparin in the short term.

### 3.4 Cytocompatibility *in vitro*

The inner layer of natural arterioles is rich in endothelial cells, which can resist thrombosis and regulate the transmission of signals and substances inside and outside the blood vessels. As an anticoagulant, heparin can avoid the clotting reaction induced by biomaterials *in vivo* as well as inhibits bacteria and cellulose adhesion. Introducing the biological function of heparin into polymer materials to achieve complete endothelization of the intima of tissue engineered vascular stent as soon as possible is an important content of the research on tissue engineered artificial blood vessels.

**FIGURE 5:**
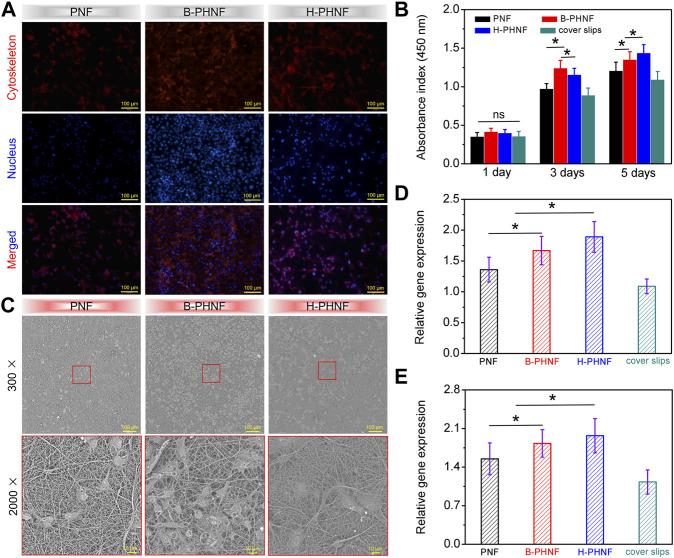
**(A)** Fluorescence microscopy images of HUVECs grown onto PNF, B-PHNF, and H-PHNF with labeling of cytoskeleton (red) and nucleus (blue) after 3-day culture, respectively; **(B)** CCK-8 assay of the proliferation viability of HUVECs cultured on PNF, B-PHNF, and H-PHNF for 1, 3, and 5 days culture, respectively; **(C)** SEM images of HUVECs grown onto PNF, B-PHNF, and H-PHNF after 3-day culture, respectively; **(D, E)** HUVECs-related gene expression of eNOS and VEGF after 7 days of culture, respectively. (Data are representatives of independent experiments and all data are given as mean ± SD, *n* = 5; ∗*p* < 0.05).

The proliferation of HUVECs grown onto PNF, B-PHNF, and H-PHNF after 3-day culture were evaluated, respectively (Figures 5A–C). It can be found from the fluorescence microscopy images in Figure 5A, the number of HUVECs in both B-PHNF group and H-PHNF groups were more than PNF group. As shown in Figures 5A,B, although the absorbance of the B-PHNF group was significantly higher than that of the H-PHNF group, the morphology of HUVECs in the H-PHNF group was significantly better than that in the B-PHNF group, and a large number of pseudopods were appeared. Figure 5C shows the growth morphology of HUVECs after culturing on the grafts for 3 days. It can be clearly observed that the number of endothelial cells adhered to B-PHNF is significantly higher than that of other groups, while HUVESs adhered to H-PHNF possessed abundant lamellipodia and filopodia, which indicates that the grafts loaded with heparin have almost no cytotoxicity and can support the adhesion and proliferation of endothelial cells. Furthermore, we selected two marker genes, eNOS and VEGF, to determine the functionality of HUVECs using reverse transcription polymerase chain reaction (RT-PCR) after 3 days of culture. H-PHNF markedly promoted HUVECs-related gene expression *in vitro* (Figures 5D,E). The H-PHNF showed a synergistic promotive effect of heparin and matching microstructure on rapid endothelization and upregulated the expression of vascular endothelial cell-related genes.

### 3.5 Patency after abdominal aorta implantation of rats

**FIGURE 6:**
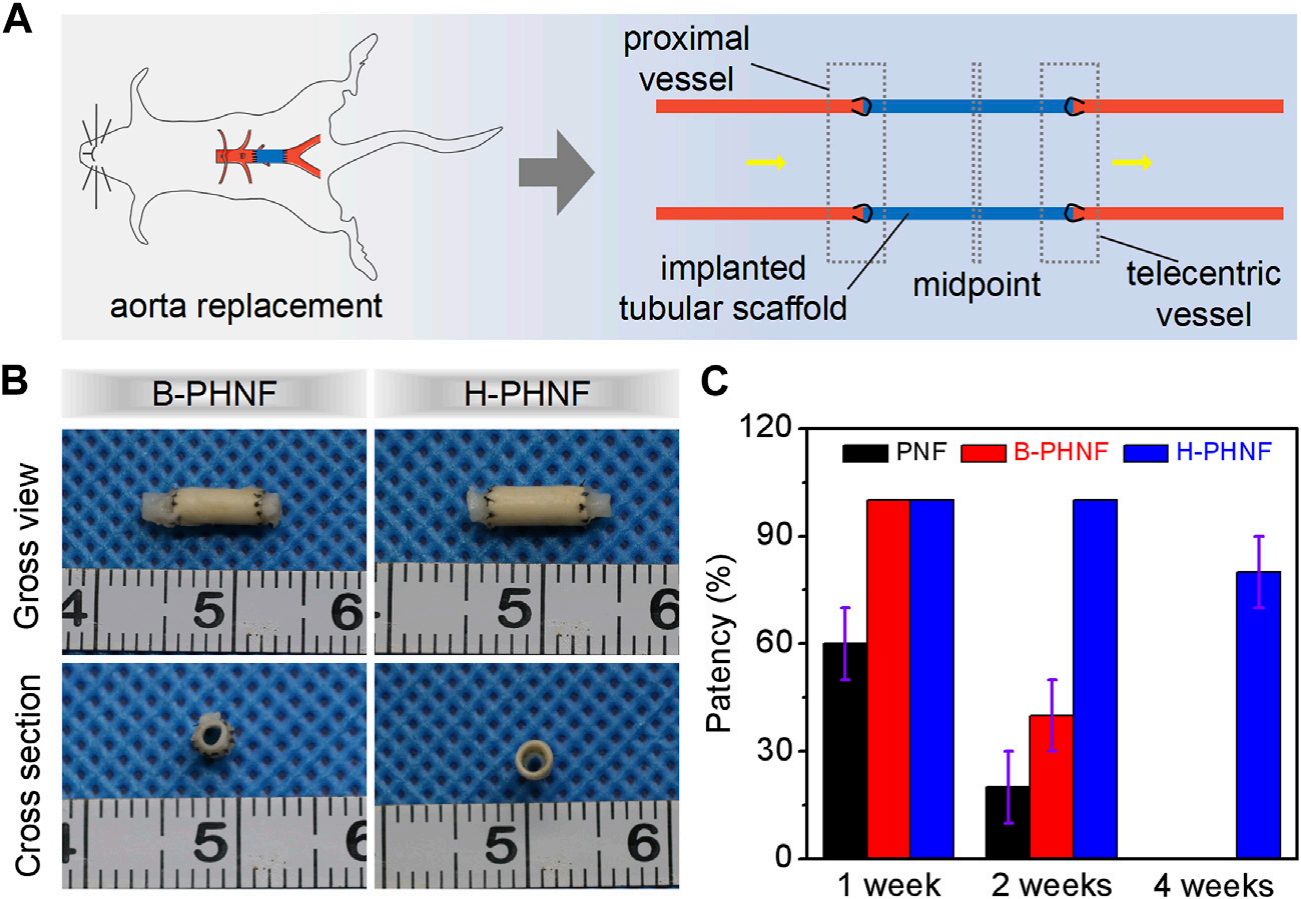
**(A)** Schematic diagram of nanofibers tubular grafts implanted into rats’ abdominal aorta via end-to-end anastomosis; **(B)** Microscopical images of the surfaces and cross-sections of the retrieved B-PHNF, H-PHNF, and its neighboring native blood vessel after implantation for 4 weeks; **(C)** Patency rate statistics for each of electrospun tubular grafts at each time point (*n* = 6).

We prepared grafts via homogeneous emulsion blending and electrospinning, followed by replacing isometric left abdominal aorta of SD rats with B-PHNF and H-PHNF by end-to-end anastomosis, respectively (Figure 6A). Nanofibers tubular grafts were retrieved for overall assessment 4 weeks after surgery. Macroscopically, the retrieved tubular graft maintained its white color and opaque texture, which can be easily distinguished from the native blood vessel that had semi-transparent appearance. More importantly, the surface of the endovascular lumen of unobstructed tubular grafts were smooth and there were no thrombus (Figure 6B). Tubular graft and native blood vessel had comparable diameters and fused well to form integrated tissue at the suture site (Figure 6B). The patency rate were detected after 1 week, 2 weeks, and 4 weeks transplantation, respectively, and the results are shown in Figure 6C. After 1 week transplantation, all the samples in both B-PHNF and H-PHNF groups remained unobstructed, while only 60% patency rate of PNF group was noted. With the increase of transplantation time, three tube of B-PHNF were blocked in the second week. In the fourth week, one tube was blocked in the H-PHNF group, and five tubes of both PNF and B-PHNF were blocked, respectively. Compared with the other groups, the H-PHNF had a longer-lasting release of heparin, which had leed to the obvious effect of inhibiting thrombus formation. This is mainly due to the technology that promotes more uniform mixing of PEEUU and heparin. Ultimately, H-PHNF exhibited stronger ability to blocks the rapid release of heparin molecules than B-PHNF.

### 3.6 Histological assessments after implantation *in situ*

**FIGURE 7:**
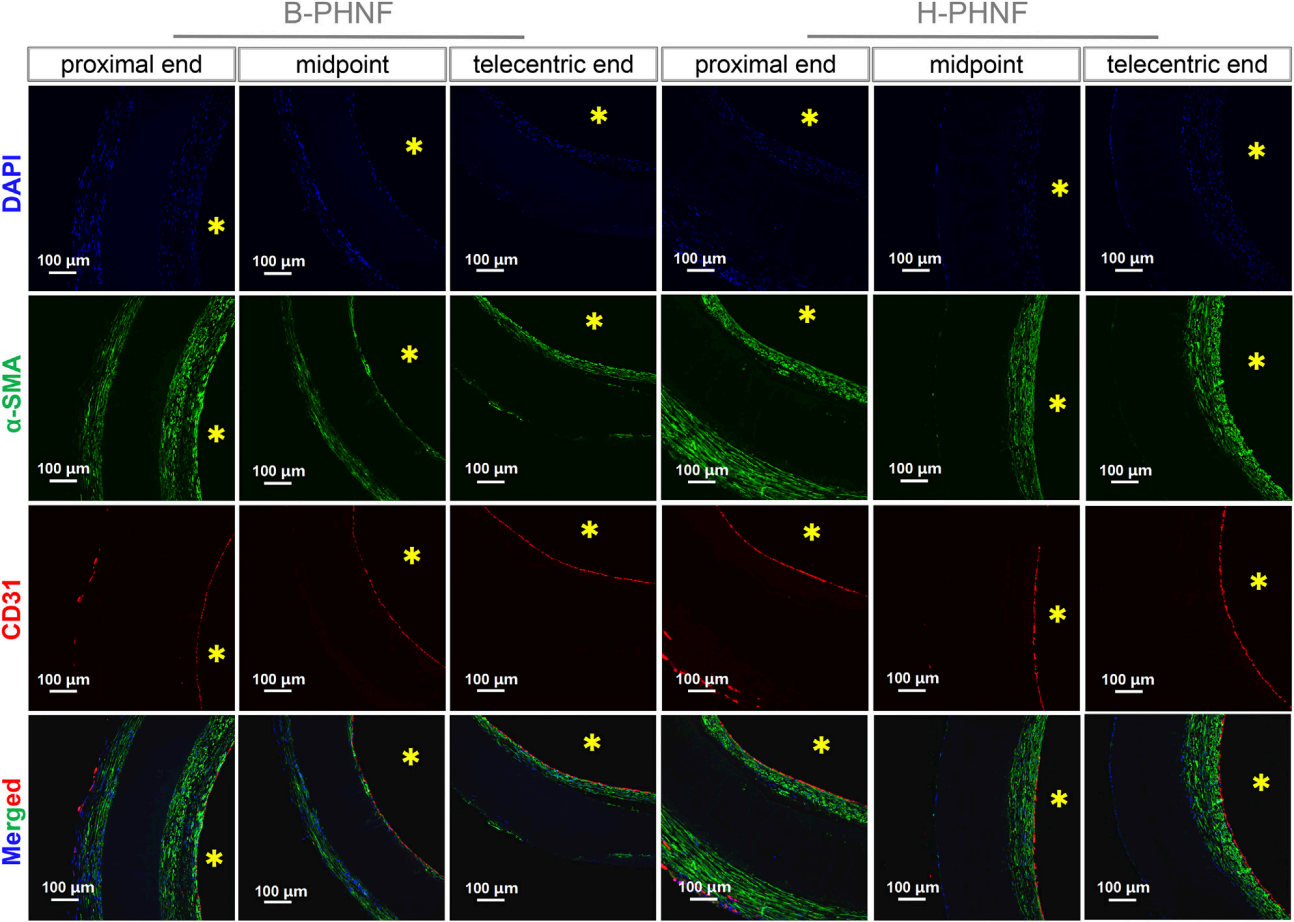
Immunofluorescent staining of cells nucleus (blue), α-SMA positive cells (green), and CD31 positive cells (red) in cross-sections of B-PHNF and H-PHNF in proximal end, midportion, and telecentric end after 4 weeks of implantation, respectively. Cell nuclei were stained with DAPI in blue, yellow * stands for lumen site.

As shown in Figure 7, the cross-sections of B-PHNF and H-PHNF in proximal end, midportion, and telecentric end after 4 weeks of implantation were stained against CD31 and α-SMA antibodies and immunofluorescent imaged to demonstrate the presence of endothelial cells and smooth muscle cells, respectively. Both B-PHNF and H-PHNF had the apparent expressions of α-SMA after implantation for 4 weeks. Compared with B-PHNF, the regenerative SMCs layer in the lumen of H-PHNF was more analogous in SMCs layer thickness, no matter in proximal end, midportion, and telecentric end, which is mainly due to the uniform distribution of heparin in H-PHNF.

The intimal layer had positive expression of endothelial cells and smooth muscle cells. The internal wall of both B-PHNF and H-PHNF lumen were completely covered by an endothelial layer composed of monolayered and uniformly distributed endothelial cells (Figure 7). No thrombosis occurred within the lumen of grafts after 4 weeks. Taken together, these data indicated that hybrided heparin contributed to enhanced anticoagulant capacity, conferred rapid endothelization, and limited intimal hyperplasia.

**FIGURE 8:**
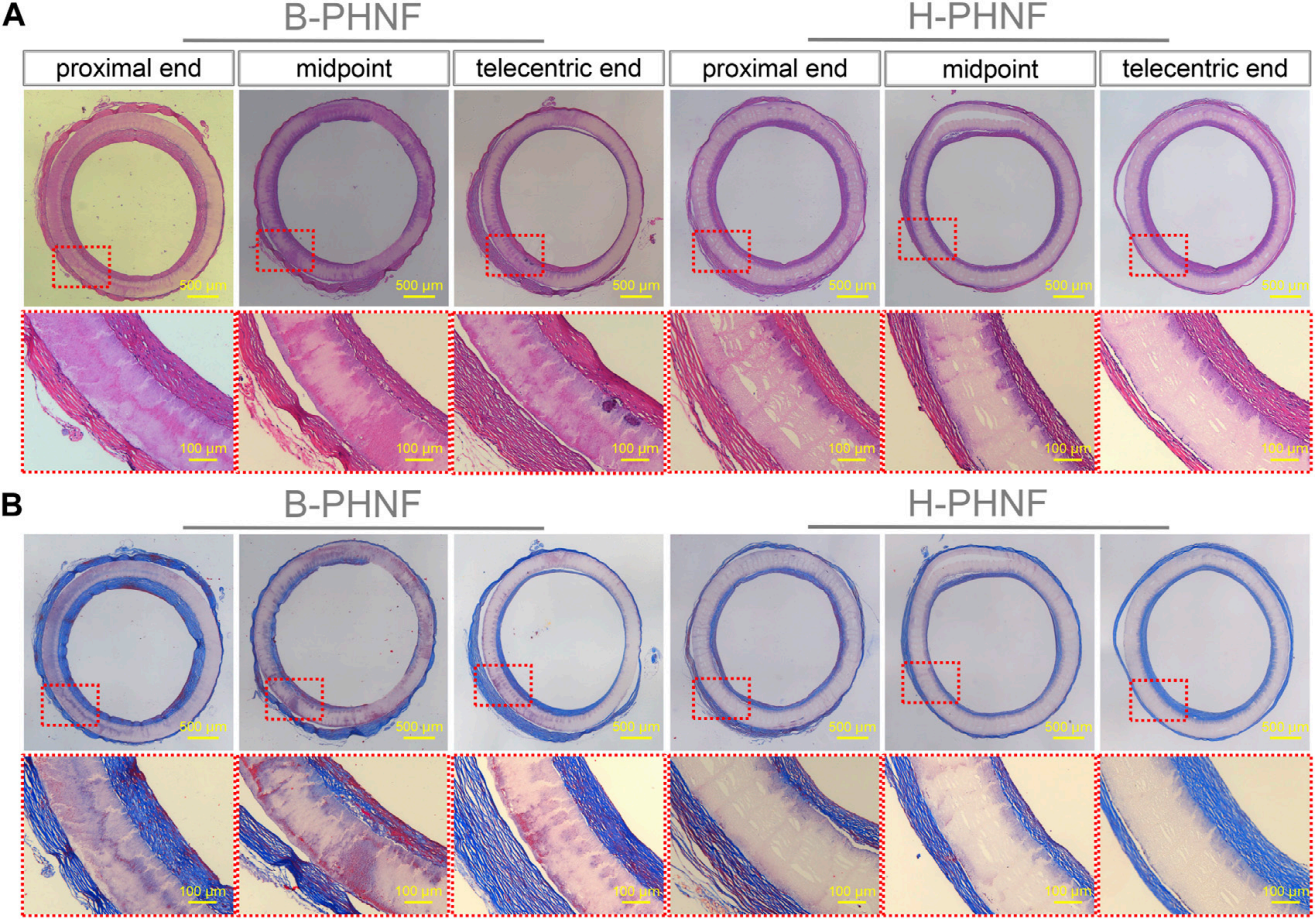
**(A)** H&E staining at three different locations, namely, proximal end, midpoint, and telecentric end of transverse section of B-PHNF and H-PHNF for 4 weeks implantation, respectively; **(B)** Masson’s trichrome staining at three different locations, namely, proximal end, midpoint, and telecentric end of transverse section of B-PHNF and H-PHNF for 4 weeks implantation, respectively.

The results of H&E and Masson’s trichrome staining at proximal end, midpoint, and telecentric end of transverse section of B-PHNF and H-PHNF for 4 weeks implantation are shown in Figure 8. H&E and Masson’s trichrome staining were used to observe the lumen area, neo-tissue formation and wall thickness of the patent grafts (Zhang Yan et al., 2022). The values of lumen diameter of proximal end, midpoint, and telecentric end in H-PHNF group were 1.98 ± 0.07 mm, 1.98 ± 0.10 mm, and 1.98 ± 0.09 mm. There was no big difference among these groups, while they were smaller than that of the B-PHNF group (1.92 ± 0.11 mm, 1.96 ± 0.08 mm, and 1.93 ± 0.13 mm for proximal end, midpoint, and telecentric end, respectively) (Figure 8A). The quantitative data is shown in Table 2. In addition, neo-tissue was observed on the luminal surface no matter in B-PHNF group or H-PHNF group. Moreover, there was discontinuous neo-tissue coverage detectable at the midpoint in B-PHNF group, while continuous neo-tissue coverage detectable at in H-PHNF group. Both H&E and Masson’s trichrome staining showed that the structure of the implanted tubular grafts were composed of a large amount of radially circumaxial oriented collagen in both inner layer and outer layer, which may be secreted by the smooth muscle layer. Therefore, we speculate that the main source of SMCs encapsulating tubular grafts is these smooth muscle tissues are mainly from the autovascular tissue migrated from the anastomosis (Figure 8B). All in all, the migration of SMCs also indicates that tubular grafts may have vascular functions such as relaxation and contraction in the later stage.

**TABLE 2:** The lumen diameter at proximal end, midpoint, and telecentric end of B-PHNF and H-PHNF for 4 weeks implantation, respectively.

Samples	Proximal end (mm)	Midpoint (mm)	Telecentric end (mm)
B-PHNF	1.92 ± 0.11	1.96 ± 0.08	1.93 ± 0.13
H-PHNF	1.98 ± 0.07	1.98 ± 0.10	1.98 ± 0.09

**FIGURE 9:**
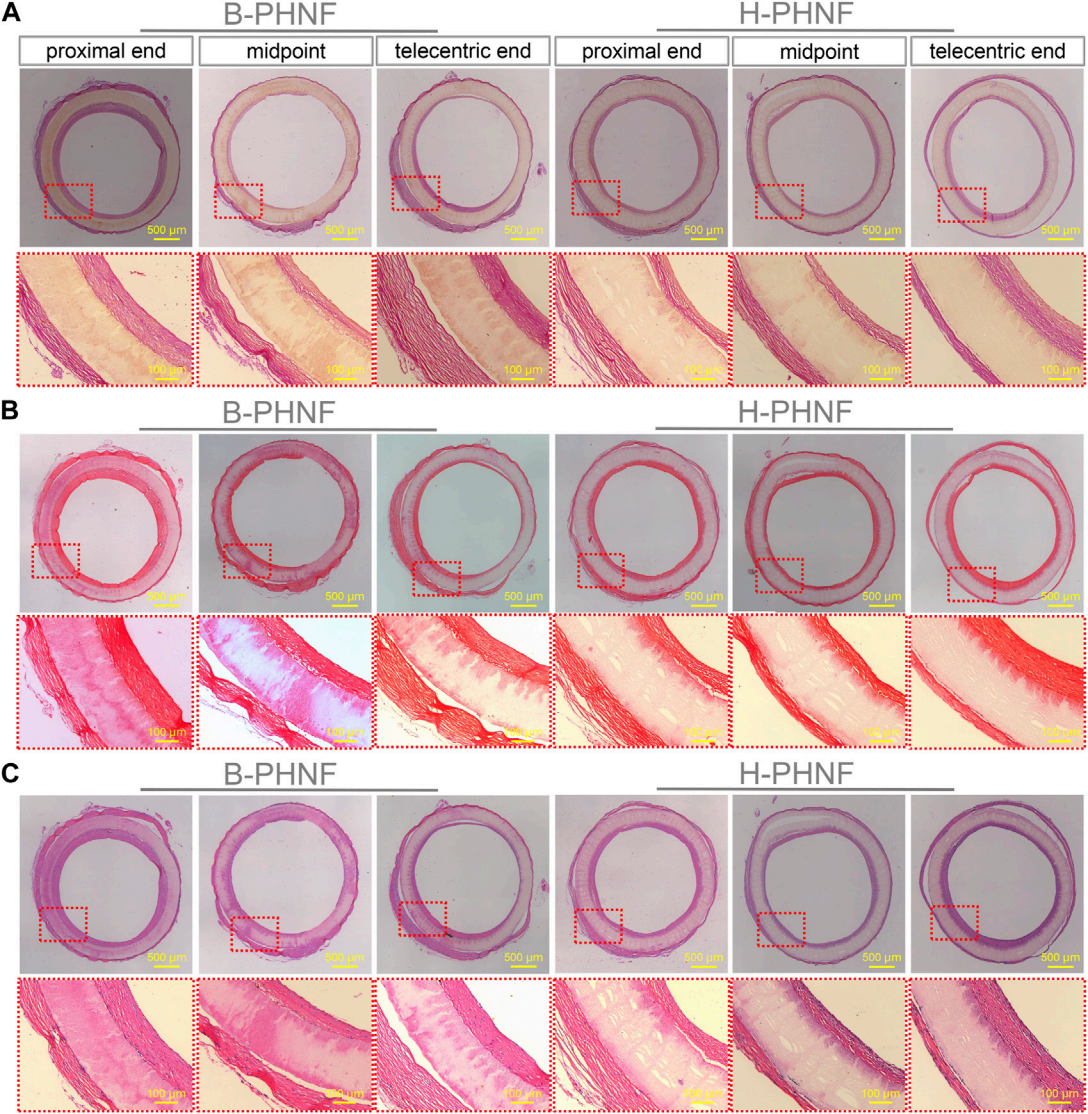
**(A)** Van Gieson, **(B)** Saffron O, and **(C)** Von Kossa staining at three different locations, namely, proximal end, midpoint, and telecentric end of transverse section of B-PHNF and H-PHNF for 4 weeks implantation, respectively.

Van Gieson staining showed that large amounts of elastic fibers were secreted in both the inner wall and outer wall of the artificial blood vessel, and elastic fibers was arranged along the circumference of the tubular grafts (Figure 9A). Elastin is an important guarantee of vascular elasticity and compliance, while elastic fibers mainly provides mechanical support. When the tubular grafts were implanted in the body to guide the orientation regeneration of smooth muscle cells, elastic fibers-oriented deposition was also achieved, which enhanced the radial mechanics of the tubular grafts and prevented the occurrence of aneurysms.

Another important substance in the natural extracellular matrix is mucopolysaccharide (GAG), a negatively charged straight-chain carbohydrate composed alternately of hexose carboxylic acids and hexose amine groups. GAG can not only form bond and regulate growth factors and cytokines, but also inhibit the decomposition of protease, and plays a positive role in the adhesion, migration, proliferation and differentiation of endothelial cells. Safranin O staining showed proteoglycan distribution on both outer and inner surfaces of tubular grafts (Figure 9B). Similarly, GAG was evenly distributed in the lumen of H-PHNF with a approximate thickness of GAG deposition, no matter proximal end, midpoint, or telecentric end, which is mainly due to the uniform distribution of heparin in H-PHNF, and that heparin is one of the main factors that stimulate the secretion of GAG in host cells.

Calcific degeneration remains a major obstacle facing the translation of tissue-engineered vascular grafts for arterial repair. The reasons that cause calcification are complex, including the elasticity of the material, the acid degradation products, and the hardness of the material. To check whether calcification had occurred after 4 weeks implantation, Von Kossa staining of transverse section of the explanted tubular graft was performed (Figure 9C). The results showed that no calcification was detected in the explanted tubular graft. Some previous studies have shown that heparin can inhibit the differentiation of vascular smooth muscle into osteoblasts in the environment as well as inhibit intracellular mineral deposition. In addition, the nanofibers with heparin homogeneous distributions promote sustained ECM secretion by host cells, which inhibits calcification due to the degradation of collagen.

## 4 Conclusion

In summary, anti-acute thrombosis cues of anticoagulant molecule heparin were incorporated into a nanofibers tubular graft (H-PHNF) of great compliance match with the native blood vessel by homogeneous emulsion blending and electrospinning. Structure and component modification confer to moderate wettability, matched mechanical and sustained drug release profile, and reliable blood compatibility of the obtained H-PHNF. H-PHNF showed prominent ability to promote endothelial cells growth and maintained patency without acute thrombosis formation in abdominal aorta implantation of rats *in situ*. This strategy may bring cell-free small-diameter synthetic vascular grafts toward clinical application.

## Data Availability

The original contributions presented in the study are included in the article/Supplementary Material, further inquiries can be directed to the corresponding authors.
