# Supplementary material for: Nanofibers with homogeneous heparin distribution and protracted release profile for vascular tissue engineering
Source: Front Bioeng Biotechnol. 2023 Jun 22;11:1187914. doi: 10.3389/fbioe.2023.1187914 (PMC10324977; doi:10.3389/fbioe.2023.1187914)
Supplement: Supplementary file 1 [file DataSheet1.doc]

Nanofibers with Homogeneous Heparin Distribution and Protracted Release Profile for Vascular Tissue Engineering

Hongmei Zhang ^b, #^, Qilu Zhang ^c, #^, Juan Du ^b,*^, Tonghe Zhu ^a, b^, Dian Chen ^d, *^, Feiying Liu ^e, *^, Yang Dong ^a, *^

*^a^ Department of Orthopedics Surgery, Shanghai Sixth People's Hospital Afffliated to Shanghai Jiao Tong University School of Medicine, 600 Yishan Rd., Shanghai 200233, P.R. China*

*^b^ School of Chemistry and Chemical Engineering, Shanghai University of Engineering Science, 333 Longteng Rd., Shanghai 201620, P.R. China*

*^c^ School of Textiles and Fashion, Shanghai University of Engineering Science, 333 Longteng Rd., Shanghai 201620, P.R. China*

*^d^ Department of Cardiothoracic Surgery, Shanghai Children’s Medical Center, Shanghai Jiao Tong University School of Medicine, 1678 Dongfang Rd., Shanghai, 200127, P.R. China*

*^e^ School of Biotechnology, East China University of Science and Technology, 130 Meilong Rd., Shanghai 200237, P.R. China*

**1 Supplementary experimental section**

**1.1 Cells culture *in vitro***

Human umbilical vein endothelial cells (HUVECs) were obtained from Shanghai Cell Bank of Chinese Academy of Sciences (Shanghai, China) and cultured with growth medium consists of dulbecco’s modified eagle medium (DMEM), 10% fetal bovine serum and 1% penicillin/streptomycin. Before cell seeding, the PNF, B-PHNF, and H-PHNF nanofibers mats were cut into discs with a diameter of 14.0 mm, then placed into 24-well plates one by one, following covered with sterilized stainless rings, respectively. Each sample was sterilized in 75% ethanol for 12 h and then washed with PBS three times, finally irradiated with ultraviolet for 2 h and socked in growth medium for incubation overnight. HUVECs were seeded at a density of 8.0 × 10^3^ cells/well, and the culture medium was maintained to be replaced every 2 days.

The cell viability of HUVECs were tested by using the Cell Counting Kit-8 (CCK-8). The cells were cultured in PNF, B-PHNF, and H-PHNF nanofibers mats for 1 day, 3 days, and 5 days, respectively. When the set culture time point, the well plate was taken from the CO_2_ incubator, and then a series of operations as removal medium, wash samples in well with PBS, adding 360 μL DMEM medium and 40 μL CCK-8 solution into the lucifugal well plate in sequence. After 4 h incubation in CO_2_ incubator, the liquid in the well plate was removed, following added 400 μL of dimethyl sulfoxide (DMSO). Then the above well plate was placed in 37 ℃ shaker for 30 min. The OD value was measured with a microplate reader at a wavelength of 450 nm.

Then, the proliferous HUVECs were fixed with 4% paraformaldehyde and dehydrated by gradient ethanol (10%, 30%, 50%, 60%, 75%, 80%, 90%, and 100%) on the 3rd day and the morphology carried out using a scanning electron microscope (SEM, Phenom XL, Netherlands) operating with sputter gold plating for 35 s at 5 mA at an accelerating voltage of 10 kV, respectively. Additionally, the cytoskeleton (red) and nucleus (blue) were stained with rhodamine labeled phalloidin and 4’,6-diamidino-2-phenylindole dihydrochloride (DAPI) after 3 days culture, respectively, according to previous staining operation. The samples were observed under the TS100 fluorescence microscope (Nikon, Japan).

After 7 days of culture, HUVECs were lysed by adding cell lysate, and then the expression of endothelium-related genes was detected by quantitative reverse transcription-polymerase chain reaction (qRT-PCR). The experimental steps of qRT-PCR mainly include the extraction of total ribonucleic acid (RNA), reverse transcription, quantitative PCR, and result processing. The primer sequences used in this study were listed in Table 2. The gene expression level of each targeted gene was detected by the 2^-ΔΔCT^ method, and the glyceraldehyde-3-phosphate dehydrogenase (GAPDH) was selected as the reference gene. Each qRT-PCR was carried out using at least five different parallel samples.

**Table 2.** Primer sequences used for qRT-PCR.

| **Name of the primer** | **Primer sequence (5’-3’)** |
| --- | --- |
| H-GAPDH-S | GGAAGCTTGTCATCAATGGAAATC |
| H-GAPDH-A | TGATGACCCTTTTGGCTCCC |
| H-eNOS-S | TGTTTGTCTGCGGCGATGT |
| H-eNOS-A | GTGCGTATGCGGCTTGTCA |
| H-VEGF-S | GGAGGGCAGAATCATCACGA |
| H-VEGF-A | GCTCATCTCTCCTATGTGCTGG |

**1.2 Whole blood clotting time test**

First, all samples (PNF, B-PHNF, and H-PHNF) were pre-incubated with PBS for 2 h in 48-well plates. Then, PBS solution was changed with 10 μL CaCl_2_ solution (0.1 M). After adding 100 μL fresh bloods to each sample, the blood clotting was activated. All samples (including tissue culture plate (TCP) were incubated at room temperature for 1, 5, 15, 25, 35, and 45 min. At the end of each time point, 1 mL of distilled water was added to the wells for another 5 min. Finally, 100 μL solution from each well (n = 5) was transferred to a 96-well plate to measure the absorbance at 540 nm.

**1.3 Plasma recalcification test**

First, all samples were added 500 μL of platelet-poor plasma (PPP) and incubated at 37 ℃ for 60 min in 24-well plates. Then 100 μL of PPP of every well was transferred to a 96-well plate and another 100 μL of CaCl_2_ solution (0.025 M) was added into the well. The whole blood clotting kinetics was monitored by measuring the absorbance at 405 nm at 37 ℃ (every 30 s for 45 min). TCPs exposed to PPP with and without CaCl_2_ were used as positive control and negative control, respectively. Five parallel experiments were proceeded to calculate the mean absorbance at each time point.

**1.4 Hemolysis test**

Healthy red blood cells (HRBCs) were obtained according to pre-treatment procedures in our previous reports for hemolysis assay. In brief, HRBCs were obtained by centrifuging the fresh blood (1200 r/min for 10 min), followed by washing the precipitates with PBS 5 times to completely remove the serum. The HRBCs were diluted 10 times with PBS before hemolysis assay. The diluted HRBCs (0.2 mL) mixed with PBS solution (10 mL) were added into a centrifugal tube with its bottom covered with nanofibers tube s (2.0 mm inner diameter, 5.0 mm length, 0.3 mm wall thickness), respectively. The diluted HRBCs (0.2 mL) were mixed with 10 mL Milli-Q water (abbreviated water (+), as a positive control) and 10 mL normal saline (abbreviated 0.9%NS (-)) (as a negative control) in centrifugal tubes for comparison. After a gentle shaking, all samples were incubated at 37 °C for 2 h. Then all the HRBC suspensions were taken away carefully and centrifuged at 2000 rpm/min for 5 min. The absorbance at 545 nm of the supernatant (hemoglobin) was determined by Lambda 25 UV-Vis spectrophotometer (Perkin Elmer, USA). Hemolysis rate (HR) was defined as Eq. (1):

HR (%) = (A_t_-A_n_)/(A_p_-A_n_)×100% (1)

where, A_t_, A_p_, and A_n_ stand for the absorbency of the experimental sample, the positive control and the negative control, respectively. The mean and standard deviation of the triplicate centrifugal tubes for each sample were calculated.

**1.5 Platelet adhesion test**

Platelet-rich plasma (PRP, 2×10^7^ platelets/mL) was obtained by centrifugation from Fresh New Zealand white rabbit blood at 1200 rpm for 10 min at room temperature. Round samples (diameter is about 14 mm) tailored from nanofibers mats were placed into 24-well plates individually and sterilized with 75% ethanol immersion for 2 h and rinsed with deionized water three times, respectively. PRP (500 µL/well) was added onto the surface of samples. After 2 h of incubation at 37 °C with mild shaking, all the samples were gently rinsed with deionized water to wash away non-attached platelets. Then, the platelets deposited on the surface were fixed in 4% paraformaldehyde for 2 h, finally dehydrated with gradient ethanol (30%, 50%, 70%, 80%, 90%, 95%, 100%) following dried at room temperature. The platelets adhered on the surfaces of nanofibers were sputter-coated with gold for SEM observation. The number of adherent platelets were determined by detecting the activity of lactate dehydrogenase (LDH Release Assay Kit, Beyotime, Nantong, China) present after cell lysis as previously described.

**1.6 Histological analysis**

The tissue was fixed with neutral formaldehyde solution and sliced in paraffin. After that, the samples were cut to 6 μm thick sections and three parts (proximal end, midpoint, telecentric end) stained by hematoxylin & eosin (H&E), Masson’s trichrome staining, Van Gieson, Saffron O and Von Kossa following standard protocols.

Immunofluorescence staining was performed on the sections using CD31 (1:100; Abcam) and α-SMA (1:100; Abcam) antibodies to visualize the regenerated endothelial cells (ECs) and smooth muscle cells (SMCs), respectively.
